# Skin-Derived Precursors against UVB-Induced Apoptosis via Bcl-2 and Nrf2 Upregulation

**DOI:** 10.1155/2016/6894743

**Published:** 2016-08-22

**Authors:** Jianqiao Zhong, Li Li

**Affiliations:** ^1^Department of Dermatology, West China Hospital, Sichuan University, Chengdu, Sichuan 610041, China; ^2^Department of Dermatology, The Affiliated Hospital of Luzhou Medical College, Luzhou, Sichuan 610041, China

## Abstract

Bcl-2 and Nrf2 are critical factors in protecting cells against UVB-induced apoptosis. Hair-follicle-bulge stem cells could resist ionization through Bcl-2 upregulation. Skin-derived precursors (SKPs) dwelling on the bulge may be against UVB irradiation. Initially, SKPs were isolated and identified. Then, SKPs were exposed to UVB and grew in medium for 24 hours. CCK-8 assay, TUNEL, and Ki67 staining evaluated cells apoptosis/proliferation, while SA-*β*gal staining evaluated cells senescence and pH2AX immunostaining evaluated DNA damage. Meanwhile, Bcl-2, Nrf2, HO-1, Bax, and Bak expressions were assessed by qRT-PCR and western blot. Two weeks later, floating spheres appeared and were identified as SKPs. After UVB radiation, SKPs maintained spherical colonies and outnumbered unirradiated ones, showing high Ki67 expression and low TUNEL, SA-*β*gal, and pH2AX expression. Fibroblasts (FBs), however, displayed deformation, senescence, and reduction, with increased TUNEL, SA-*β*gal, and pH2AX expression. Moreover, Bcl-2 and Nrf2 mRNA expression were significantly higher than Bak and Bax in irradiated SKPs. Conversely, Bcl-2 and Nrf2 mRNA levels greatly decreased compared with Bax and Bak in irradiated FBs. Interestingly, SKPs showed higher protein levels of Bcl-2, Nrf2, and HO-1 than FBs. SKPs exert a beneficial effect on resisting UVB-induced apoptosis, which may be associated with Bcl-2 and Nrf2 upregulation.

## 1. Introduction

Cumulative Ultraviolet B (UVB) radiation can damage the skin, encourage photoaging, and even induce tumorigenesis [1, 2]. It has been shown to have a substantial impact on patients' health, but treatments remain unsatisfactory so far. Apoptosis and repair mechanisms are involved in UVB-induced DNA damage or apoptosis, in which Bcl-2 and transcription factor NF-E2-related factor (Nrf2) play pivotally protective roles [3, 4]. Both of them are critical genes to alleviate UV-associated skin damage or skin aging [3, 5, 6]. As an antiapoptotic gene, Bcl-2 belongs to bcl-2 family which can promote cell survival by blocking the transmission of proapoptotic signals, whereas proapoptotic genes like Bak and Bax accelerate apoptosis [3, 7]. Nrf2, a key regulator against UV insult, can efficiently protect skin cells from UV-induced DNA damage or apoptosis [8–10]. After exposure to UV, Nrf2 translocates into the nucleus and its signaling pathway is provoked, which further activates downstream cytoprotective genes and upregulates antioxidant enzyme heme oxygenase-1 (HO-1) to resist UVB-mediated cellular injury and eventually sustains cells survival [11]. Generally, apoptosis prevails in DNA or cell damage induced by UV radiation or ionization, which leads to gH2AX phosphorylation (pH2AX, an important biomarker of DNA damage) presence, cells reduction, senescence, and death [12–15]. However, some reports indicated that after hair-follicle-bulge stem cells (SCs) or embryonic stem (ES) cells were exposed to a certain dose of ionizing radiation (IR), cells biological activity and viability were rarely affected, let alone their differentiation or senescence, the mechanism of which was associated with Bcl-2 upregulation [16, 17]. Recently skin-derived precursors (SKPs), a population of adult stem cells, were isolated from dermis with self-renewal and multilineage differentiation capacities [18, 19]. It was demonstrated that the bulge region of hair and whisker follicle papillae may be endogenous niches for SKPs [20–22]. In view of SKPs niches, we hypothesized that SKPs may be resistant to UVB-induced DNA damage or apoptosis. In order to test this hypothesis, we isolated SKPs from mouse dermis, observed the changes of SKPs after exposure to UVB, and detected the levels of bcl-2 family and Nrf2.

## 2. Materials and Methods

### 2.1. Mice and Cells

Neonatal Balb/C mice aged 1–3 days were purchased from the Center of Experimental Animal, West China Hospital, Sichuan University, China. Murine ES cells (129/S6, SCR012, Millipore) and mouse dermal fibroblasts (FBs, BALB-5067, Cell Biologics) were given as a gift by Professor Qintong Li, College of Life Sciences, Sichuan University, China.

### 2.2. Ethics Statement

The experiment was approved by the Animal Care & Welfare Committee of West China Hospital, Sichuan University (permit number: 2011016A) and was strictly performed as per the* Guide for the Care and Use of Laboratory Animals*. The surgery was operated under intraperitoneal injection of pentobarbital anesthesia to guarantee less pain and less discomfort for animals.

### 2.3. Reagent Setup

*Wash Medium.* Wash medium was DMEM/F12 (3 : 1) containing 1% penicillin/streptomycin (Invitrogen, Carlsbad, CA, USA).

*Proliferation Medium.* Proliferation medium was DMEM/F12 (3 : 1) containing 0.1% penicillin/streptomycin, 40 ng/mL FGF2, 20 ng/mL EGF, and 2% B27 supplement (Invitrogen, Carlsbad, CA, USA).

*Basal Medium.* Basal medium was DMEM/F12 (3 : 1) containing 0.1% penicillin/streptomycin and 6% fetal bovine serum (FBS; Hyclone, Logan, UT, USA).

*Adipogenesis Inducer Solution.* Adipogenesis inducer solution was basal medium containing 2% 3-isobutyl-1-methylxanthine (IBMX) solution, 1% insulin solution, and 1% dexamethasone solution (Invitrogen, Carlsbad, CA, USA).

*Osteogenesis Inducer Solution.* Osteogenesis inducer solution was basal medium containing 1.6% BMP-2 solution, 1.2%  *β*-GP solution, 1.2% Vc solution, and 1.2% dexamethasone solution (Invitrogen/Gibco, Carlsbad, CA, USA).

### 2.4. Cell Isolation and Culture

SKPs were isolated and cultured as described [18, 23]. Dorsal skin was dissected from mouse neonates and cut into 2-3 mm^2^ pieces. After being washed three times in Hanks balanced salt solution (HBSS; Invitrogen, Carlsbad, CA, USA), dissected pieces were digested with 0.1% trypsin (Gibco, Carlsbad, CA, USA) for 30–50 min at 37°C. When tissue pieces became pale and ropey, the epidermis could be easily removed from the dermis. Subsequently, dermis pieces were mechanically dissociated with scissors and repeatedly triturated in wash medium with a 1,000 *μ*L pipette tip. The supernatant was collected and the trituration was repeated until tissue pieces became thin. After the dissociated cell suspension was filtered through a 40 *μ*m cell strainer and centrifuged at 1,200 r.p.m. for 7 minutes, the pellet was resuspended in proliferation medium to an optimum density of 10,000–30,000 cells per mL of medium. Finally, cells were floatingly cultured in proliferation medium at 37°C/5% CO_2_ and used for experiments at passages 2 through 3.

At the same time, FBs were cultured as control in low-sugar DMEM (Invitrogen, Carlsbad, CA, USA) medium plus 10% FBS (Hyclone, Logan, UT, USA). Besides, ES cells grew in DMEM containing 15% ES cell-grade fetal bovine serum (Gemini Bio-Products, Gemini, Calabasas, CA, USA), supplemented with LIF (Millipore, Billerica, MA, USA), sodium pyruvate, 2-mercaptoethanol, and nonessential amino acids. All these cells were passaged every 3-4 days and experimentalized at 2-3 passages.

### 2.5. SKPs Identification

#### 2.5.1. Western Blot Analysis

SKPs were prepared for western blot analysis, while FBs and ES cells were collected as controls. Equal amounts of protein were resolved on 6% or 12% sodium dodecyl sulfate-polyacrylamide gel electrophoresis (SDS-PAGE). The primary antibodies used were anti-Nestin monoclonal (1 : 1,000; Millipore, Billerica, MA, USA), anti-Sox2 polyclonal (1 : 1,000; Millipore, Billerica, MA, USA), and anti-*β*-Actin polyclonal (1 : 2,000; Millipore, Billerica, MA, USA).

#### 2.5.2. Osteocytes and Adipocytes Differentiation of SKPs

For differentiation, SKPs were trypsinized and dissociated into single cells and then cultured in basal medium with B27 supplement for 3 days. Subsequently basal medium was replaced by osteocytes and adipocytes inducer solution, conditions leading to 21-day osteocytes and adipocytes differentiation, respectively. Finally, cells were stained with Alizarin Red Solution (Boao, Beijing, China) and Oil Red Solution (Yansheng, Shanghai, China) separately, additionally floating cells without inducer as control.

### 2.6. UVB Irradiation on Cells

#### 2.6.1. UVB Irradiation Dose

The dose of UVB was based on previous dermal FBs data [24] and the condition was optimized. Briefly, SKPs were cultured in a proliferation medium at 37°C and 5% CO_2_. After starvation with growth factors-free medium for 24 hours, cells were centrifuged and washed twice with PBS. Then, they were seeded and exposed to UVB with 3-4 drops of PBS. After irradiation, PBS was aspirated and replaced with proliferation medium. UVB irradiation was performed using a UVB lighter (Sigma, Shanghai, China) and cells were vertically irradiated at a distance of 20–25 cm from lighter without the lid. UVB irradiation doses were tested on a 0–50 mJ/cm^2^ range and finally fixed at 30 mJ/cm^2^ for further experiment. As a control, FBs were also operated according to the above procedure.

#### 2.6.2. Cells Viability Assay

Cell apoptosis/proliferation was assessed by CCK-8 assay, terminal deoxynucleotidyl transferase dUTP nick end labeling (TUNEL) assay, and Ki-67 immunostaining. Meanwhile, senescence-associated *β*-galactosidase (SA-*β*gal) staining and pH2AX immunostaining were, respectively, used to evaluate cell senescence and DNA damage. UVB irradiation was carried out using the same UVB lighter. The cells of SKPs and FBs were divided into four groups: SKPs irradiated group, SKPs unirradiated group, FBs irradiated group, and FBs unirradiated group. Initially, SKPs and FBs were cultured, respectively, in a proliferation medium and low-sugar DMEM medium plus 10% FBS at 37°C and 5% CO_2_. Then, after feeding with growth factors-free or 10% FBS-free medium for 24 hours, cells were plated at a density of 5 × 10^3^ cells/well in 96-well plates or 5 × 10^3^ cells/slide on glass slides and subjected or not to UVB (30 mJ/cm^2^) for 120 seconds. Subsequently, four-group cells, respectively, grew in medium containing growth factors or 10% FBS. After 24 h of incubation, cells were, respectively, determined by CCK-8 assay (Dojindo, Suite, Japan), TUNEL assay (Roche, Switzerland), SA-*β*gal staining (Invitrogen, USA), Ki67 (1 : 100; Abcam, Cambridge, UK), and pH2AX immunostaining (1: 200; Abcam, Cambridge, UK) according to the manufacturer's instructions. Positively stained cells were counted in five randomly selected fields in a blinded manner under microscope.

#### 2.6.3. Quantitative Real-Time PCR Analysis for Antiapoptotic/Proapoptotic Genes

To investigate the alteration of antiapoptotic and proapoptotic genes, we carried out quantitative real-time PCR (qRT-PCR) analysis to measure mRNA. Prior to RNA extraction, cells were also divided into four groups as mentioned above and performed in 6-well plates (inoculum density: 5 × 10^4^ cells/well). The procedures of cell culture and UVB irradiation were in the same way as described in* Cells Viability Assay*. After UVB irradiation and culture for 24 h, cells were harvested and washed twice with PBS for RNA extraction. RNA was extracted by Trizol total RNA extraction kit (Invitrogen, Carlsbad, CA, USA) and operated as instructions. Quantitative real-time PCR (qRT-PCR) was performed on iCycler iQ instrument (BioRad, Hercules, CA, USA) and *β*-Actin was used as internal reference genes. The evaluation of relative mRNA levels among four groups was performed by using the ΔΔCT method. The following primers were used: *β*-Actin: 5′-CCCATCTATGAGGGCTACGCT-3′ (forward) and 5′-AGGTGGAAGGTCGTTTACACC-3′ (reverse); Bcl-2: 5′-ATGGGGTGAACTGGGGGAGGATTG-3′ (forward) and 5′-GGCCAGGCTGAGCAGGGTCTTC-3′ (reverse); Nrf2: 5′-TCCCATTTGTAGATGACCATGAG-3′ (forward) and 5′-CCATGTCCTGCTCTATGCTG-3′ (reverse); Bax: 5′-CCGCGTGGTTGCCCTCTTCTAC-3′ (forward) and 5′-TTTCCCCTTCCCCCATTCATCC-3′ (reverse); Bak: 5′-AGCCGGGAATGCCTACGAAC-3′ (forward) and 5′-GGCCCAACAGAACCACACCA-3′ (reverse).

#### 2.6.4. Western Blot Analysis for Bcl-2, Nrf2, and HO-1 Expressions

For detection of Bcl-2, Nrf2, and HO-1 protein levels, SKPs and FBs were prepared and collected as above (shown in the part of* Quantitative Real-Time PCR Analysis for Antiapoptotic/Proapoptotic Genes*). Then, cells were, respectively, lysed in lysis buffer and protein loading buffer for total protein extraction. The proteins were subjected to western blot analysis with anti-Bcl-2 monoclonal antibody (1 : 1000; BD Biosciences, USA), anti-Nrf2 polyclonal antibody (1 : 2000, Millipore, Billerica, MA, USA), and anti-HO-1 polyclonal antibody (1 : 2000; Abcam, England). Bcl-2 and Nrf2 immunodetection on 10% or 12% SDS-PAGE revealed Bcl-2 and Nrf2 band. Besides, anti-*β*-Actin polyclonal (1 : 2000; Millipore, Billerica, MA, USA) was used as internal reference protein.

### 2.7. Statistical Analysis

All data analyses and summarizations were performed using SPSS 15.0. The differences between groups were analyzed by Student's *t*-test and *p* < 0.05 was considered significant.

## 3. Results and Discussion

### 3.1. Characterization of SKPs

**Figure 1:**
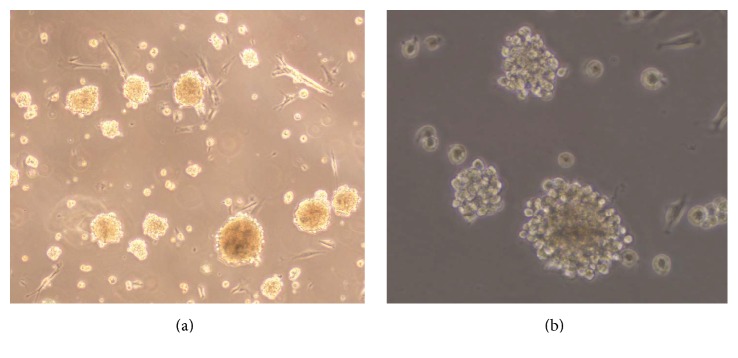
Isolation and characterization of SKPs derived from neonatal Balb/C mice. (a) Low magnification image of SKPs: after being cultured for 2 weeks in proliferation medium, a large number of SKPs grew as spheres in suspension (×200). (b) High magnification image of SKP spheres: different-sized spherical colonies exhibited round and phase bright morphology (×400).

**Figure 2:**
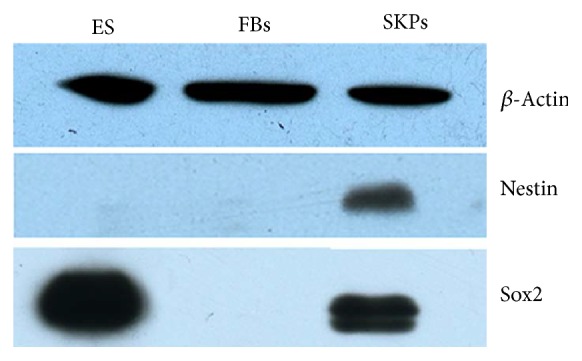
Western blot analysis for two markers expression in SKPs. *β*-Actin was used as an internal control. ES: embryonic stem cells; FBs: fibroblasts; SKPs: skin-derived precursors.

**Figure 3:**
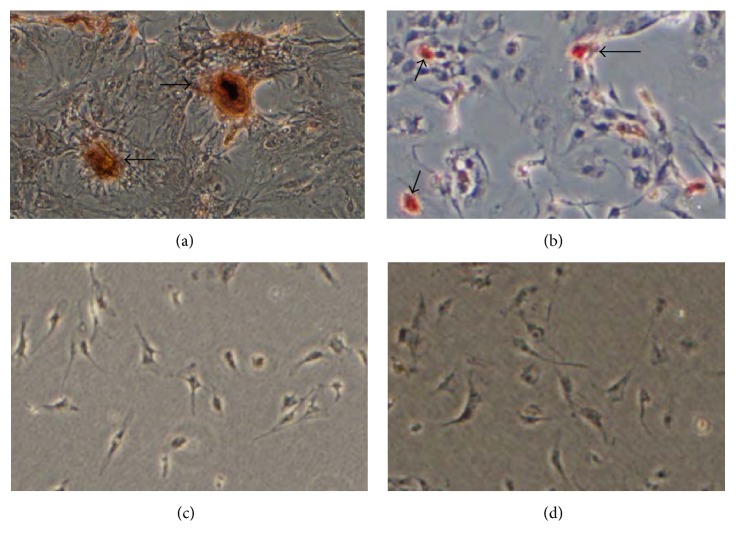
Differentiation of SKPs into osteocytes and adipocytes. (a) After being cultured for 3 weeks in osteogenesis induction medium, SKPs differentiated into bone-like cells with central aggregation and fusion, as well as multiple high-density calcium nodules which were stained yellowish red by Alizarin Red Solution (arrows indicated calcium nodules positively stained by Alizarin Red Solution). (b) After being cultured for 3 weeks in adipogenesis induction medium, SKPs differentiated into round or vacuolated cells with lipid droplets, which were in part stained bright red by Oil Red Solution (arrows indicated vacuolated cells positively stained by Oil Red Solution). (c) and (d) Both as control, after being cultured in medium without inducer, SKPs were not stained by either Alizarin Red Solution or Oil Red Solution.

In order to get SKPs, cells were separated from dermis and cultured in serum-free medium. After 2 weeks, SKPs were successfully isolated and cultured by suspension culture from the dermis of mouse. The cells expanded easily* in vitro* and displayed spherical colonies morphology (Figure 1). By western blot, characteristic expressions of SKPs-related surface markers were confirmed. SKPs spheres positively expressed Nestin and SOX2, whereas ES cells were Sox2-positive and Nestin-negative, and FBs both negative (Figure 2). After 3-week induction, SKPs could differentiate into osteocytes and adipocytes (Figures 3(a) and 3(b)); this phenomenon, however, did not occur when these cells were cultured in medium without inducer (Figures 3(c) and 3(d)). The characteristics of the above cells coincided with those of SKPs reported in documents [18–21, 25–27]. In 2001, SKPs were firstly isolated and cultured in suspension from mammalian dermis by Toma et al. with serum-free medium including EGF, FGF, and B27 [18]. After that, many researchers had also harvested these cells in a similar way [25, 26]. They altogether demonstrated that SKPs displayed round and phase bright floating spheres, had a multilineage differentiation capacity, and expressed the markers of Nestin, Sox2, Sox9, Pax3, slug, Twist1, Snail, and so forth [20, 21, 25–27]. In our previous [23] and current studies, we both successfully isolated SKP-shaped spheres from the dermis and identified them as SKPs based on the fact that these cells present the key markers such as Nestin and Sox2 and have the ability to differentiate into osteocytes or adipocyte. The above findings provide cells for the subsequent studies.

### 3.2. Resistance of SKPs to UVB-Induced Apoptosis and DNA Damage

**Figure 4:**
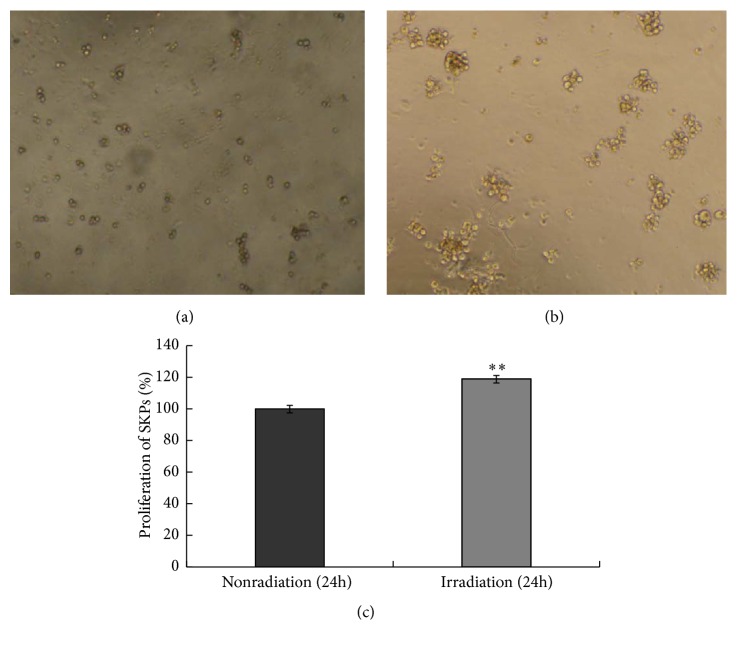
Effect of UVB irradiation on SKPs apoptosis/proliferation by CCK-8 analysis. (a) The morphology of SKPs cultured for 24 hours without UVB irradiation. (b) The morphology of SKPs cultured for 24 hours after UVB irradiation. (c) The comparison of SKPs proliferation between irradiated group and unirradiated group. *∗∗* SKPs in irradiated group (irradiation) were compared with those in unirradiated group (nonradiation), *p* < 0.01.

**Figure 5:**
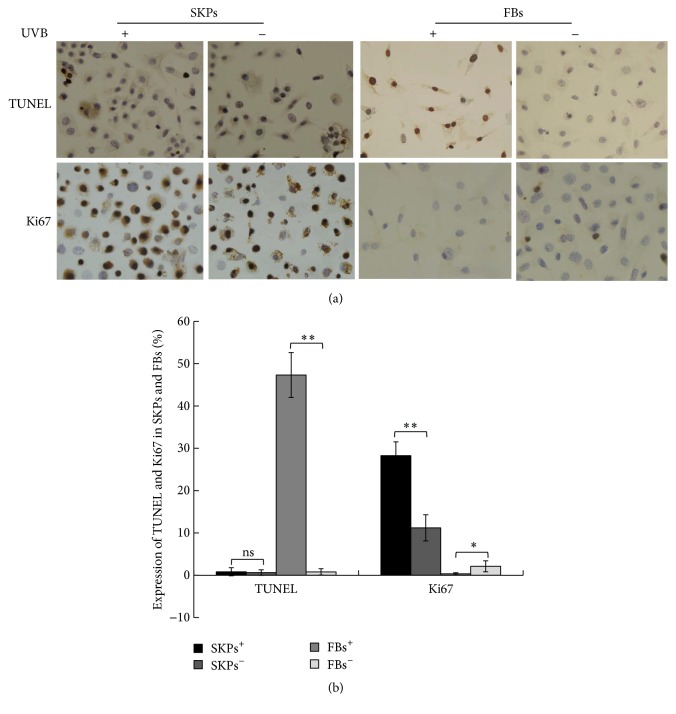
Effect of UVB irradiation on cell apoptosis/proliferation by TUNEL and Ki67 analysis. (a) Immunohistochemical analysis of Ki67-positive and TUNEL-positive expression in SKPs and FBs after UVB irradiation (magnification ×400). +: UVB irradiation; −: non-UVB irradiation. In the TUNEL assay and Ki67 immunostaining, nuclei in apoptotic cells and proliferating cells appear brown. (b) Quantitative analysis of TUNEL^+^ cells and Ki67^+^ cells in four groups. SKPs^+^: SKPs irradiated group; SKPs^−^: SKPs unirradiated group; FBs^+^: FBs irradiated group; FBs^−^: FBs unirradiated group. ^*∗*^ *p* < 0.01; ^*∗∗*^ *p* < 0.001; ns: no significance.

**Figure 6:**
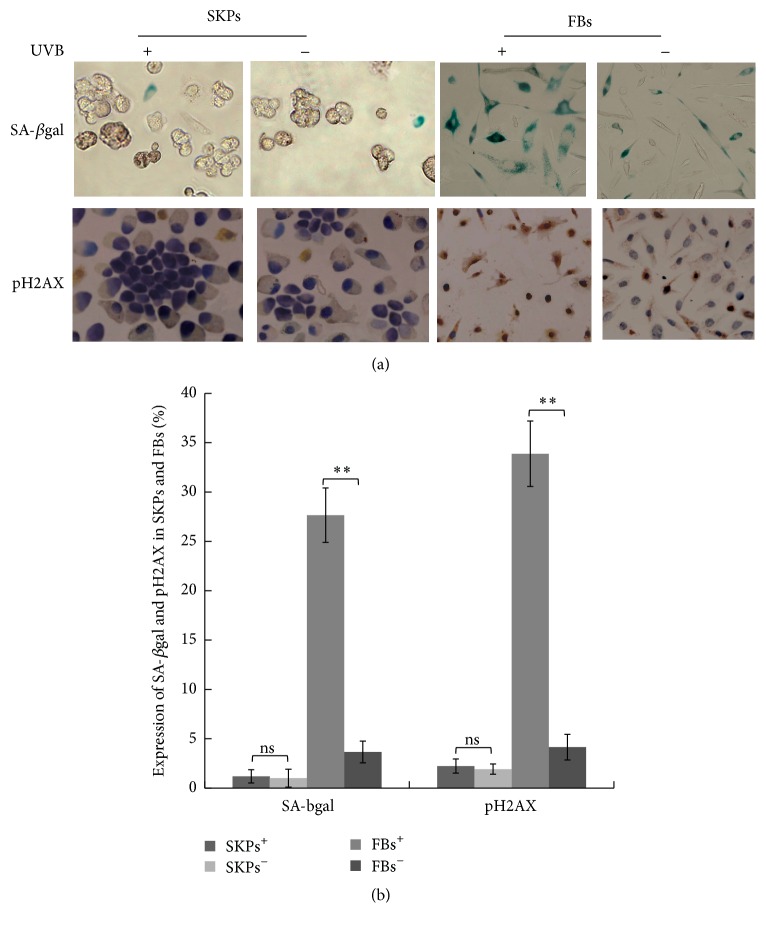
Effect of UVB irradiation on cell senescence and DNA damage by SA-*β*gal and pH2AX analysis. (a) Immunohistochemical analysis of SA-*β*gal-positive and pH2AX positive expression in SKPs and FBs after UVB irradiation (magnification ×400). +: UVB irradiation; −: non-UVB irradiation. In the SA-*β*gal staining, senescent cells were stained blue-green with no stain in nuclei, while DNA-damaged cells display brown nuclei in pH2AX analysis. (b) Quantitative analysis of SA-*β*gal^+^ cells and pH2AX^+^ cells in four groups. SKPs^+^: SKPs irradiated group; SKPs^−^: SKPs unirradiated group; FBs^+^: FBs irradiated group; FBs^−^: FBs unirradiated group. ^*∗∗*^ *p* < 0.001; ns: no significance.

**Figure 7:**
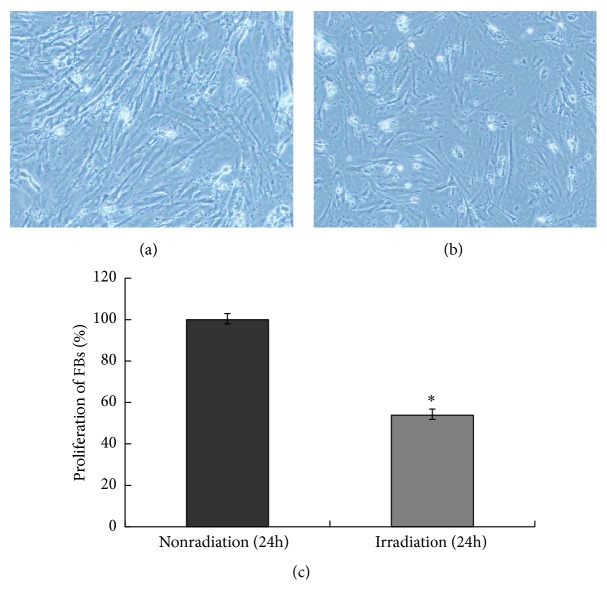
Effect of UVB irradiation on FBs apoptosis/proliferation by CCK-8 analysis. (a) The morphology of FBs cultured for 24 hours without UVB irradiation. (b) The morphology of FBs cultured for 24 hours after UVB irradiation. (c) The comparison of FBs proliferation between irradiated group and unirradiated group. *∗* FBs in irradiated group (irradiation) were compared with those in unirradiated group (nonradiation), *p* < 0.01.

In the repair process of UVB-induced cell damage, apoptosis and proliferation play important roles. To investigate the functional role of SKPs against UVB-induced apoptosis, senescence, and DNA damage, we first stained apoptotic/proliferating cells with TUNEL and Ki67, labeled aging cells by SA-*β*gal, and used special biomarker pH2AX for detecting cells with DNA damage. Subjected or not to UVB radiation, SKPs grew into germ-form or spherical cloning clusters from a single cell 24 hours later (Figures 4(a) and 4(b)), followed by a lot of Ki67-positive cells and few TUNEL, SA-*β*gal, and pH2AX labeling cells (Figures 5(a) and 6(a)). On the contrary, FBs partially showed necrosis, senescence, shrinkage, and strong refraction with round or irregular shape and a number of TUNEL, SA-*β*gal, and pH2AX positive cells were observed after UVB intervention (Figures 7(b), 5(a), and 6(a)), whereas FBs without UVB radiation grew well, displaying little necrosis and spindle morphology with few TUNEL and SA-*β*gal positive cells (Figures 7(a), 5(a), and 6(a)). Compared with SKPs unirradiated group, SKPs irradiated group apparently proliferated by 18.90% (*p* < 0.001) and Ki67^+^ cells significantly increased by 17.05% (*p* < 0.001), while only 1.02 %, 1.15 %, and 2.26 % of cells were, respectively, positive for TUNEL, SA-*β*gal, and pH2AX, which showed no statistical significance (Figures 4(c), 5(b), and 6(b)). Conversely, there was a more significant quantitative reduction of 46.12% in irradiated FBs than unirradiated ones (*p* < 0.001), followed by 47.32% TUNEL^+^ cells, 27.65% SA-*β*gal^+^ cells, and 33.85% pH2AX^+^ cells (*p* < 0.001, resp.) (Figures 7(c), 5(b), and 6(b)). These findings implied that SKPs were not only resistant to UVB-induced apoptosis and DNA damage, but also greatly proliferated after UVB irradiation in this experiment, which were to some extent inconsistent with other reports. In general, it is considered that cells after UVB radiation or IR would become versiform, injured, aged, reductive, and even apoptotic [13–15, 28, 29]. This phenomenon indeed occurred in FBs in our study, indicating the obvious reduction and deformation of FBs after a certain dose of UVB radiation. However, it did not happen to SKPs after UVB radiation. Our results, inversely, manifested that SKPs were morphologically invariant and quantitatively increased by 18.90%, highly expressing Ki67, and scarcely showing TUNEL, SA-*β*gal, and pH2AX positive expression after exposure to 30 mJ/cm^2^ UVB. It indicated that a certain dose of UVB radiation could stimulate SKPs proliferation and SKPs rarely undergo senescence, DNA damage, or apoptosis, further suggesting that SKPs have the effect of resistance to UVB. Similar results reported by Sokolov et al. conformed our findings [16, 17, 30]. They found that the biological activities and viabilities of ES cells, hair-follicle-bulge SCs, or mesenchymal SCs were rarely affected after being exposed to a certain dose of IR (1 Gy or 5 Gy); in other words, these cells rarely displayed differentiation or senescence but proliferation. Besides, Shim et al. recently also found that dermal stem cell-derived conditioned medium could attenuate UV-induced cell damage [31], which further confirmed our study outcomes. Naturally, a deep consideration was drawn on the mechanisms of UVB-induced SKPs proliferation. Hence, we have detected antiapoptotic/proapoptotic genes and antiapoptotic proteins which were involved in UVB-induced apoptosis or damage.

### 3.3. Upregulation of Bcl-2, Nrf2, and HO-1 Encourages SKPs against UVB-Induced Apoptosis

**Figure 8:**
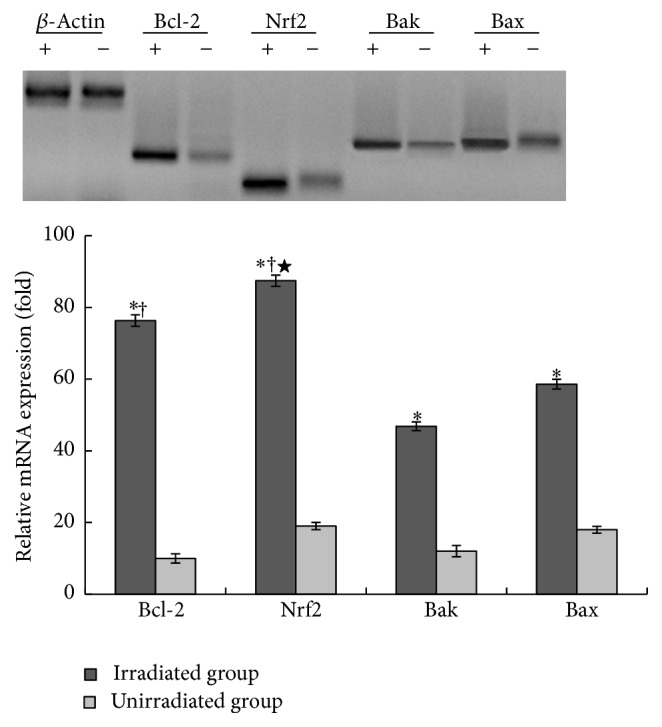
Expression of the apoptotic and antiapoptotic genes in UVB-irradiated SKPs. +: UVB irradiation; −: non-UVB irradiation. *∗* Four genes' mRNA expressions in irradiated group were, respectively, in comparison with those in unirradiated group, *p* < 0.01; † The mRNA expressions of Bcl-2 and Nrf2 were separately in comparison with those of Bax and Bak in irradiated group, *p* < 0.01; ★ Nrf2 mRNA expression was in comparison with Bcl-2 in irradiated group, *p* < 0.01.

**Figure 9:**
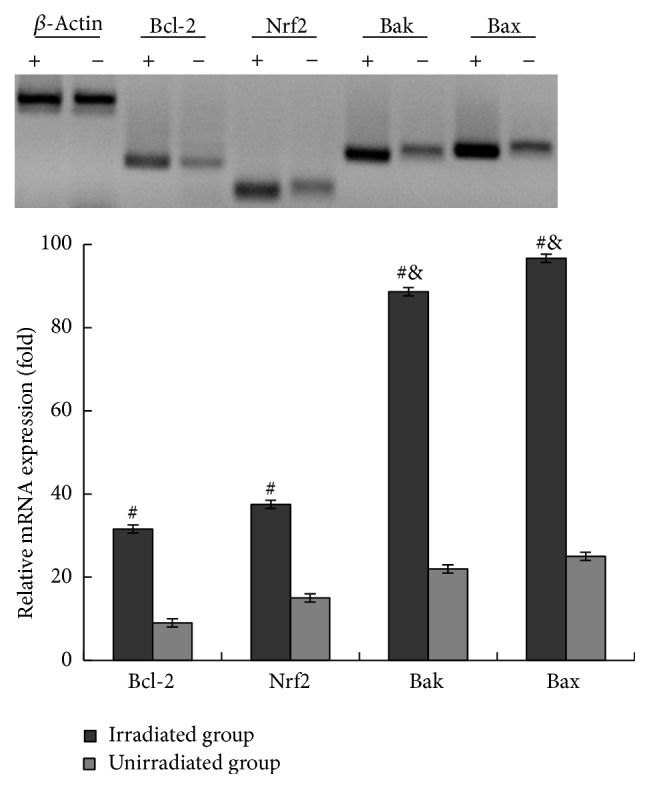
Expression of the apoptotic and antiapoptotic genes in UVB-irradiated FBs. +: UVB irradiation; −: non-UVB irradiation. # Four genes' mRNA expression levels in irradiated group were, respectively, in comparison with those in unirradiated group, *p* < 0.01; & The mRNA expression levels of Bax and Bak were separately in comparison with that of Bcl-2 and Nrf2 in irradiated group, *p* < 0.01.

**Figure 10:**
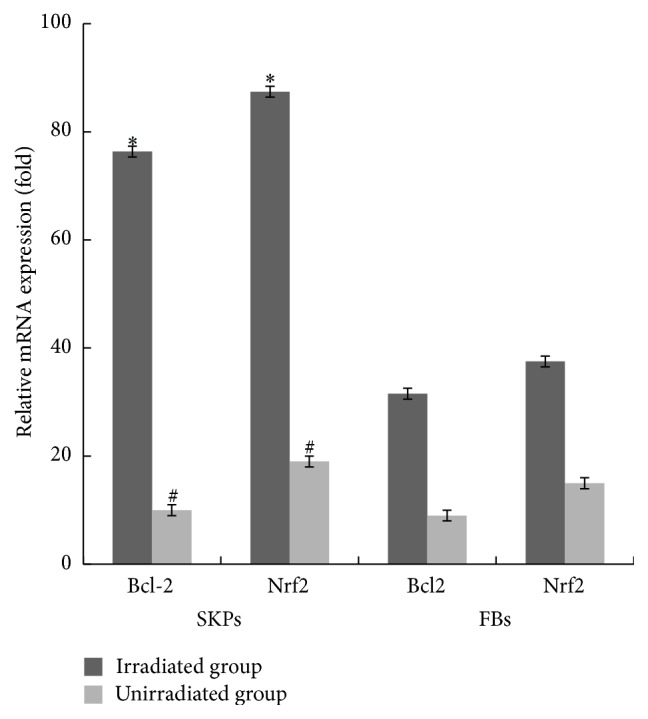
Quantification of Bcl-2 and Nrf2 mRNA expression in SKPs and FBs by qRT-PCR. *∗* The mRNA expression levels of Bcl-2 and Nrf2 in SKPs were, respectively, in comparison with those in FBs in irradiated group, *p* < 0.01; # Bcl-2 and Nrf2 mRNA expression levels in SKPs were, respectively, in comparison with those in FBs in unirradiated group, *p* < 0.05.

**Figure 11:**
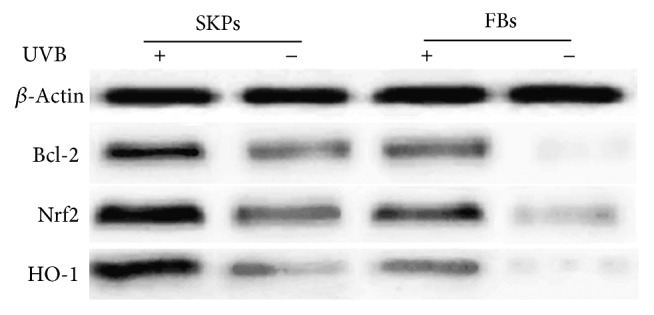
Western blot analysis for Bcl-2, Nrf2, and HO-1 protein expressions in SKPs and FBs. +: UVB irradiation; −: non-UVB irradiation; *β*-Actin was used as an internal control.

To determine the mechanisms of SKPs against UVB-induced apoptosis, we performed qRT-PCR and western blot in SKPs for analysis of antiapoptotic or proapoptotic gene and antiapoptotic protein levels. Agarose gel electrophoretogram showed that Bcl-2, Nrf2, Bak, and Bax genes expression in SKPs or FBs with UVB irradiation was positively stronger than those without irradiation (Figures 8 and 9). Accordingly, mRNA expression levels of those genes in irradiated cells obviously ascended compared with unirradiated cells (*p* < 0.01) (Figures 8 and 9). Bcl-2 and Nrf2 mRNA levels, however, appeared to be much higher than Bak and Bax in irradiated SKPs (*p* < 0.01), particularly Nrf2 (*p* < 0.001) (Figure 8). On the other hand, the mRNA levels of Bcl-2 and Nrf2 were apparently lower than those of Bax and Bak in FBs (*p* < 0.001) (Figure 9). More importantly, mRNA and protein levels of Nrf2 and Bcl-2 were significantly higher in SKPs than in FBs, especially in irradiated SKPs (*p* < 0.001) (Figures 10 and 11). To further verify whether UVB could activate Nrf2, the downstream protein HO-1 expression was detected. By western blot assay, UVB-irradiated SKPs exhibited apparently elevated HO-1 protein expression compared with irradiated FBS (Figure 11), suggesting that UVB irradiation could increase Nrf2 expression, promote Nrf2 activation, and induce Nrf2-mediated HO-1 upregulation. AS SKPs highly expressed Bcl-2 and Nrf2 after UVB exposure, it indicated that SKPs proliferation or SKPs antiapoptosis might be mediated by the upregulation of Bcl-2 and Nrf2 in our study. Bcl-2 and Nrf2, as two crucial antiapoptotic factors, are extremely important in confronting IR or UVB-induced cell damage, which can protect cell from apoptosis and promote cell survival [3, 10, 32]. Our results showed that Bcl-2, Nrf2, Bax, and Bak gene expression in SKPs increased after UVB irradiation, more significantly with the former two. Instead, the genes of Bcl-2 and Nrf2 were weakly expressed in irradiated FBs, while Bax and Bak were strongly expressed. Interestingly, the mRNA and protein levels of Bcl-2 and Nrf2 in SKPs tended to be higher than those in FBs after UVB irradiation. The outcomes from our study indicated that the increased protein expression of Bcl-2, Nrf2, and HO-1 in SKPs may be responsible for SKPs resisting UVB-induced apoptosis or damage. As a result, these findings are helpful in clarifying the reasons of SKPs proliferation and FBs reduction after UVB irradiation. Similarly, Sotiropoulou PA et al. also found that Bcl-2 expression was higher after hair-follicle-bulge SCs were radiated by IR [17]. Thus they concluded that an alternative mechanism for hair-follicle-bulge SCs resistance to IR-induced cell death was closely related to high expression of Bcl-2 gene and Bcl-2 was quite critical in hair-follicle-bulge SCs against IR-induced apoptosis. At the same time, it was considered that Nrf2 could strongly reduce UVB-induced cytotoxicity and control selective protection of keratinocytes from UVB-mediated apoptosis through activation of cytoprotective genes and upregulation of detoxification enzymes/antioxidant proteins like HO-1 [9, 10, 33]. These foregoing studies were in accordance with our findings, suggesting that the reasons for SKPs resistance to UVB-induced apoptosis may be correlated with the upregulation of Bcl-2 and Nrf2. Therefore, we speculate that the possible mechanism of SKPs against UVB-derived apoptosis is that upregulation of Bcl-2 and Nrf2 expression accelerates DNA repair and protects cells from damage or death; ultimately cells survive.

## 4. Conclusions

From our study, we demonstrated that SKPs have a distinctive effect on resisting UVB-induced apoptosis or damage. Moreover, it confirmed that the capacity of SKPs against UVB-induced apoptosis or damage is connected with Bcl-2 upregulation and Nrf2 activation as well as Nrf2-mediated HO-1 elevation. Our findings may reveal a novel characteristic of SKPs, contribute to a better understanding of the susceptibility of tissues to UVB-induced cell damage or aging, and lead to the development of new strategies for photodamage/photoaging and skin cancer. Thus, additional studies* in vivo* to further confirm our* in vitro* outcomes will be needed in the future.
